# Combination therapy of mitochondria-targeted antioxidants and polyphenols for early intervention in Huntington’s disease

**DOI:** 10.17179/excli2024-8025

**Published:** 2025-01-20

**Authors:** Anuradha Acharya, Sukriti Vishwas, Sachin Kumar Singh

**Affiliations:** 1 School of Pharmaceutical Sciences, Lovely Professional University, Phagwara, Punjab, India

## ⁯⁯⁯⁯⁯

A neurodegenerative disease that causes significant movement deficits, cognitive decline, and psychiatric problems, Huntington's disease (HD) is debilitating and eventually fatal. About 5 to 10 out of every 100,000 people globally are affected, and symptoms typically start to show between the ages of 30 and 50 (Pringsheim et al., 2012[4]). A hereditary mutation in the Huntingtin (HTT) gene results in an unusually long polyglutamine chain in the huntingtin protein, which is the cause of HD. This mutation causes progressive brain cell loss and injury by interfering with the survival and function of neurones, especially in regions such as the cortex and striatum. Current treatments for HD mostly control symptoms rather than decreasing the disease's course, despite substantial research efforts. The lack of treatments that can change how the illness progresses emphasizes how urgently new strategies that deal with its underlying causes are needed. Oxidative stress and mitochondrial dysfunction are major factors in HD, which causes neuronal energy deficits and, eventually, cell death (Gu et al., 1996[2]). By interfering with mitochondrial function, the mutant huntingtin protein (mHTT) raises dangerous reactive oxygen species (ROS) and initiates cell death.

Antioxidants specially designed to collect within mitochondria and neutralize ROS at its source are MitoQ (mitoquinone) and SkQ1 (Smith et al., 2012[5]; Vishwas et al. 2020[6]). By enhancing neuronal survival, lowering oxidative damage, and restoring mitochondrial function, these substances have demonstrated promise in preclinical settings. For example, in animal models, MitoQ has been shown to penetrate the blood-brain barrier and reduce neurodegeneration (Ghosh et al., 2010[1]). However, naturally occurring substances called polyphenols, which are present in plants, have strong anti-inflammatory, neuroprotective, and antioxidant qualities. The capacity of polyphenols including resveratrol, curcumin, and epigallocatechin gallate (EGCG) to alter certain signaling pathways linked to neurodegeneration has been investigated (Mancuso et al., 2006[3]). For instance, curcumin suppresses the aggregation of misfolded proteins and lowers inflammation, whereas resveratrol activates SIRT1 and promotes mitochondrial biogenesis.

### Hypothesis and rationale

Early intervention for HD may benefit from a combination therapy that uses polyphenols and antioxidants that target the mitochondria. Addressing both aberrant signaling pathways and mitochondrial dysfunction - two important aspects in the course of HD - is the goal of this dual-targeted approach. While antioxidants that are unique to mitochondria are especially good at neutralizing ROS within mitochondria, offering localized protection, polyphenols, which are recognized for their systemic antioxidant qualities, can lessen oxidative stress. Additionally, this combination strategy may aid in restoring brain energy balance, which is essential for preserving neuronal health, by improving mitochondrial bioenergetics and biogenesis. Additionally, polyphenols contribute to lowering the cellular toxicity linked to protein aggregation by preventing the aggregation of mHTT. Additionally, by lowering cytokine production and microglial activation, the anti-inflammatory qualities of antioxidants and polyphenols may assist regulate neuroinflammation. Polyphenols and mitochondrial antioxidants are intriguing candidates for a complete treatment approach to reduce early HD pathogenesis because of their combined effects.

Overall it can be stated that polyphenols and mitochondria-targeted antioxidants together could offer a potential treatment approach for HD that targets several pathological processes. This method of early intervention may help patients live better lives by slowing the advancement of their diseases. To confirm this theory and open the door to novel HD therapy possibilities, more investigation is required, including thorough preclinical and clinical investigations.

## Conflict of interest

The authors declare no conflict of interest.
